# Applying Natural Language Processing Techniques to Map Trends in Insomnia Treatment Terms on the r/Insomnia Subreddit: Infodemiology Study

**DOI:** 10.2196/58902

**Published:** 2025-01-09

**Authors:** Jack A Cummins, Daniel J Gottlieb, Tamar Sofer, Danielle A Wallace

**Affiliations:** 1 Manchester Essex Regional High School Manchester, MA United States; 2 Division of Sleep and Circadian Disorders Departments of Medicine and Neurology Brigham and Women’s Hospital Boston, MA United States; 3 Division of Sleep Medicine Harvard Medical School Boston, MA United States; 4 VA Boston Healthcare System Boston, MA United States; 5 Department of Biostatistics Harvard T.H. Chan School of Public Health Boston, MA United States; 6 CardioVascular Institute (CVI) Beth Israel Deaconess Medical Center Boston, MA United States; 7 Division of Sleep and Circadian Disorders Department of Medicine Brigham and Women’s Hospital Boston, MA United States

**Keywords:** insomnia, natural language processing, NLP, social media, cognitive behavioral therapy, CBT, sleep initiation, sleep disorder, easly awakening, sleep aids, benzodiazepines, trazodone, antidepressants, melatonin, treatment

## Abstract

**Background:**

People share health-related experiences and treatments, such as for insomnia, in digital communities. Natural language processing tools can be leveraged to understand the terms used in digital spaces to discuss insomnia and insomnia treatments.

**Objective:**

The aim of this study is to summarize and chart trends of insomnia treatment terms on a digital insomnia message board.

**Methods:**

We performed a natural language processing analysis of the r/insomnia subreddit. Using Pushshift, we obtained all r/insomnia subreddit comments from 2008 to 2022. A bag of words model was used to identify the top 1000 most frequently used terms, which were manually reduced to 35 terms related to treatment and medication use. Regular expression analysis was used to identify and count comments containing specific words, followed by sentiment analysis to estimate the tonality (positive or negative) of comments. Data from 2013 to 2022 were visually examined for trends.

**Results:**

There were 340,130 comments on r/insomnia from 2008, the beginning of the subreddit, to 2022. Of the 35 top treatment and medication terms that were identified, melatonin, cognitive behavioral therapy for insomnia (CBT-I), and Ambien were the most frequently used (n=15,005, n=13,461, and n=11,256 comments, respectively). When the frequency of individual terms was compared over time, terms related to CBT-I increased over time (doubling from approximately 2% in 2013-2014 to a peak of over 5% of comments in 2018); in contrast, terms related to nonprescription over-the-counter (OTC) sleep aids (such as Benadryl or melatonin) decreased over time. CBT-I–related terms also had the highest positive sentiment and showed a spike in frequency in 2017. Terms with the most positive sentiment included “hygiene” (median sentiment 0.47, IQR 0.31-0.88), “valerian” (median sentiment 0.47, IQR 0-0.85), and “CBT” (median sentiment 0.42, IQR 0.14-0.82).

**Conclusions:**

The Reddit r/insomnia discussion board provides an alternative way to capture trends in both prescription and nonprescription sleep aids among people experiencing sleeplessness and using social media. This analysis suggests that language related to CBT-I (with a spike in 2017, perhaps following the 2016 recommendations by the American College of Physicians for CBT-I as a treatment for insomnia), benzodiazepines, trazodone, and antidepressant medication use has increased from 2013 to 2022. The findings also suggest that the use of OTC or other alternative therapies, such as melatonin and cannabis, among r/insomnia Reddit contributors is common and has also exhibited fluctuations over time. Future studies could consider incorporating alternative data sources in addition to prescription medication to track trends in prescription and nonprescription sleep aid use. Additionally, future prospective studies of insomnia should consider collecting data on the use of OTC or other alternative therapies, such as cannabis. More broadly, digital communities such as r/insomnia may be useful in understanding how social and societal factors influence sleep health.

## Introduction

Insomnia is a condition characterized by self-reported difficulty in falling asleep or staying asleep, with accompanying distress or impairment [1]. Insomnia symptoms can be chronic or acute [2]. Approximately 10%-30% of the population is estimated to experience either chronic or acute insomnia, with insomnia more common among women and older adults [3,4]. One prospective cohort study reported an insomnia incidence of 13.9%—of those, 37.5% developed chronic insomnia over a 5-year period [5].

People experiencing insomnia may report decreased quality of life and daily functioning [6,7], have higher injury risk [8], and may seek multiple treatment options to improve their sleep and address insomnia symptoms [9]. Insomnia treatment approaches have also changed over time. Hypnotics used to be the primary treatment option for insomnia until behavioral treatment options such as cognitive behavioral therapy for insomnia (CBT-I) were shown to be highly clinically effective [10]. Alternative herbal remedies for insomnia, such as herbal teas to induce somnolence, have also been popular throughout history [11]. Trends in social mention of insomnia treatment options may track with changes in approaches for treatment in the medical community, but they may also reflect additional community-based approaches due to limitations in access to treatment or cultural preferences. These community-based approaches, such as using herbal teas and supplements, may not be reflected in medical records or drug prescription rates.

The language used by individuals seeking and obtaining insomnia treatment may be heterogeneous and may differ from standard medical terminology for the treatment provided [10]. For example, one of the components of CBT-I consists of sleep hygiene, which addresses routines and environmental factors that influence sleep initiation and maintenance [12]; however, the term “hygiene” may connote a negative association, implying the person seeking help has “dirty” sleep habits [13]. Understanding the language that people use in digital insomnia-related communities and the sentiment attached to treatment terms may be useful for health practitioners as they work to understand how to discuss symptoms and treatment options with their patients, as well as for sleep researchers engaged in data collection. Mapping trends in language around insomnia may also provide insights helping to interpret the use of related terms over time and may identify temporal patterns in the use of medication and other therapies for insomnia.

Digital health-related message boards can be important sources of frank discussion and community and provide insight into beliefs, practices, and language. Reddit is one such digital community with medically related subreddits; prior analyses of these subreddits have used well-established, reliable, natural language processing (NLP) techniques such as bag of words (BOW), regular expressions (RE), and Valence Aware Dictionary and Sentiment Reasoner (VADER) to better understand patient or community experiences [14-16]. For example, Low et al [17] analyzed data from Reddit mental health communities using various NLP techniques to identify changes in various mental health–related subreddits during the COVID-19 pandemic. However, these techniques have not been previously applied to the r/insomnia subreddit. The r/insomnia subreddit may be a particularly rich source to examine insomnia using NLP techniques because sleeplessness is commonly self-treated outside of a medical setting. In a 2022 American Academy of Sleep Medicine (AASM) survey (n=2010 US adults), 64% of respondents reported using a substance to help them sleep, with 23% reporting the use of prescription medications and 41% reporting the use of an over-the-counter (OTC), herbal, or other substance; the use of a sleep aid was most prevalent among respondents aged 18-54 years [18]. Comments on r/insomnia, an anonymous forum, may be a resource for understanding self-treatment behavior and the use of prescription or nonprescription substances. We characterize the language used in a digital discussion board of insomnia using an NLP analysis of the r/insomnia subreddit to measure the discussion frequency of insomnia-related substances and to identify the sentiment (positive or negative) when substances are mentioned.

## Methods

### Overview

Our goals were to measure the discussion frequency of insomnia-related substances and to identify the sentiment. Thus, we applied a set of NLP methods that (1) extract common words from text, (2) identify substance-related treatment terms (relying on human recognition of some terms), and (3) assign sentiment to sentences containing such terms. First, the r/insomnia data were collected from the Pushshift dataset. Next, comments were converted to word counts using a BOW model. Using this BOW model, the most common nonstop words were identified, and the insomnia treatment–related terms were selected. The BOW approach was chosen because (1) it is a simple and efficient method and (2) summarizing and quantifying the popularity or frequency of treatment terms was a primary goal; in this way, disadvantages of BOW, such as loss of word order, were not deemed to be problematic for our purposes. REs were then applied to quantify the use of these terms in the subreddit over time. The RE approach was chosen because (1) the percentage of comments containing a term is an effective way to gauge the popularity or interest in a term, and (2) it is able to find all comments that contain a pattern of letters rather than just individual words (like the BOW method). For example, the RE pattern “sleep” would also capture related words with the same root, such as “asleep,” “sleep,” “sleeping,” and “sleeplessness.” Finally, sentiment analysis was applied on the raw comments (not word counts; Figure S1 in Multimedia Appendix 1). Sentiment analysis was chosen because, in addition to measuring interest in these terms and treatments, the goal of this study was to gauge how social media users felt about the terms and treatments. In general, the average sentiment of comments containing the treatment term is expected to reflect the sentiment toward that treatment.

### Data Source

All subreddit comments from the history of r/insomnia [19] in 2008 up to the end of 2022 were obtained from the top 20,000 subreddits Pushshift dataset, which is a publicly available dataset [20,21]. All comments from the r/insomnia subreddit from 2008 to 2022 were included in each data analysis step. Data analysis was performed with Python (version 3; Python Software Foundation), and statistical testing was performed with R (version 4.4.0; R Foundation for Statistical Computing). Analyses that were summarized by year used comments dated up to December 31, 2022. All methods of analysis detailed below refer to individual comments (responses, not including the original post). If a thread of conversation included multiple comment responses from different commenters or the same commenter, then each comment within the thread would be counted as a separate comment in the total. This study did not meet the criteria for human participant research as defined by Mass General Brigham Human Research Office policies and Health and Human Services regulations set forth in 45 CFR 46. The data were obtained from a publicly available anonymous digital forum.

### Identifying Treatment-Related Words Using a BOW Model

A BOW model, which extracts text features without regard to the order, was created using the *gensim* package [22]. A BOW model counts the number of occurrences of each word in a comment (excluding “stop words,” such as “the” and “a”). To create the BOW model, we first preprocessed all comments from the r/insomnia subreddit using the gensim simple preprocessor function, which removed symbols and numbers and converted the comments into lists of words. The natural language toolkit “wordNetLemmatizer” was used to convert words to their dictionary forms. For example, the word “dogs” is simplified to “dog,” but the word “benzos” would not be shortened to “benzo” because it is an abbreviation and is not included in the dictionary. Stop words were removed from comments. Words used in fewer than 15 comments were removed. After the BOW model was created, the 1000 words with the highest comment frequency (number of comments that contain the word at least once) were retrieved. The list of 1000 words for the r/insomnia subreddit was reviewed manually by one of the authors (DAW) and reduced to words based on relevance to insomnia medication or insomnia-related treatment terms. The words are referred to henceforth as the “BOW list.” To identify additional brand or generic drug names for the treatment terms that referred to a medication, we also performed an extensive search using the Epocrates drug reference platform in addition to Google and Wikipedia. Terms that referred to the same treatment, such as generic versus brand names of medications (eg, quetiapine and Seroquel), were combined in deriving term counts (Figure S1 in Multimedia Appendix 1).

### Using REs to Identify Textual Patterns in the Data

In addition to BOW, we used RE to identify patterns in the text by identifying and counting comments that contained each of the selected words of interest from the BOW list within r/insomnia. The RE searches were set as case insensitive. We used Matplotlib [23] to visualize the percentage of r/insomnia comments containing each of the prespecified words each year. The percentage of comments containing a word each year was calculated as the total number of comments containing the specified word divided by the total number of comments in the subreddit extracted from that year, not accounting for authorship. To standardize the data and account for changes in total r/insomnia comment volume over time, all data are reported as the percentage of total annual comments that contain the specified term (ratio of comments containing the term relative to the total annual comment volume). The analysis focused on the years 2013 to 2022 because comment volume was low prior to 2013. For some related words, search terms were combined so that comments containing any of multiple words (eg, “CBT,” “CBTI,” or “hygiene”) were counted together in the analysis.

### Sentiment Analysis of Insomnia Treatments

**Figure 1:**
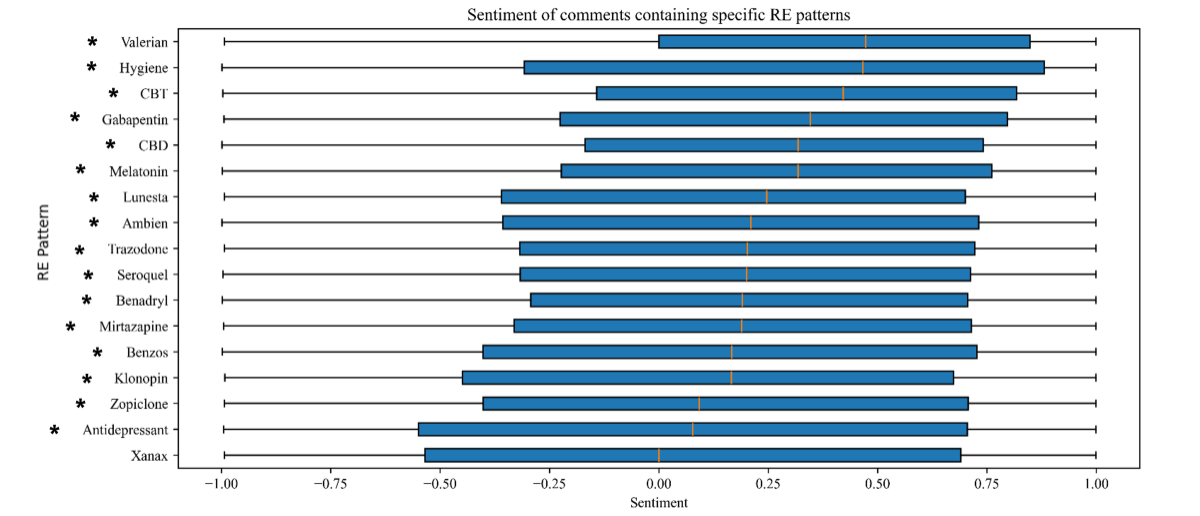
Boxplot of sentiment of the treatment and medication terms identified in r/insomnia comments after randomly selecting 1 comment per author. The blue boxes depict from the first quartile to the third quartile, with the median marked by an orange line. The black lines extend to minimum and maximum values. Asterisks mark terms with sentiment significantly different from 0 in 1-sample Wilcoxon signed-rank test after Bonferroni correction for multiple testing (*P*<.003). CBD: cannabidiol; CBT: cognitive behavioral therapy; RE: regular expression.

Sentiment analysis was also applied to identify the overall emotional tone of the text containing the terms of interest. Sentiment analysis was performed using the VADER [20], a reliable and commonly used lexicon and rule-based tool specifically trained on and developed for use with social media data [22-25]; VADER has been applied in numerous prior health-related analyses of Reddit data [26-28]. VADER calculates the polarity of text on a normalized scale from –1 to 1, 1 being the most positive sentiment for a comment and –1 being the most negative sentiment for a comment. The resulting sentiment values from comments containing insomnia-related treatment terms were compared. To test whether sentiment by treatment term category significantly differed from 0, comments with treatment terms were subset to randomly select 1 comment per author (for independence; Figure 1), and sentiment tested to be different from 0 using 1-sample Wilcoxon signed rank tests (2-sided). Bonferroni correction was used to correct for multiple testing, and results (*P*<.003) were considered statistically significant.

### Ethical Considerations

Ethical concerns exist with the use of internet and social media data and should be considered prior to and throughout the data analysis process. Although our project received a research exemption, we believe it is important to mention some of the ethical issues around using Reddit data. The data we used in this analysis are publicly available. However, social media can also potentially contain identifying information if names or other identifiers are contained within the data. Reddit is considered an anonymous site where users create their own usernames or pseudonyms [29] compared to other social media sites, where people may use their real names; we also do not mention usernames or provide quotes from comments to protect the anonymity of commenters [30] and present aggregated results. Additionally, the r/insomnia subreddit has a sizeable community base compared to smaller subreddits, where commenters may know each other [30]. Given these considerations, we believe that our analysis aligns with current ethical guidelines around the use of internet data [31].

## Results

There were 340,130 comments from the r/insomnia subreddit. Only data from the years 2013 to 2022 were included in the trend analyses due to sparse data (less than 2500 comments per year) through 2012.

### Insomnia Subreddit Growth Over Time

The number of comments per year in the r/insomnia subreddit grew steadily from 306 comments in 2011 to 86,623 comments in 2022 (Table S1 and Figure S2 in Multimedia Appendix 1). The number of unique commenters also grew from 155 commenters in 2011 to 14,866 commenters in 2022 (Table S1 in Multimedia Appendix 1). The number of unique authors commenting on the subreddit increased every year except for 2021, when there was a 2.5% decrease in unique authors. The number of comments also increased every year. The subreddit grew the greatest in 2019, with 20,942 (79.5%) more comments and 4319 (88.5%) more unique commenters compared to 2018. The rate of increase in number of comments and commenters began to plateau from 2020 to 2022.

### Patterns in Treatment and Medication Term Use

**Figure 2:**
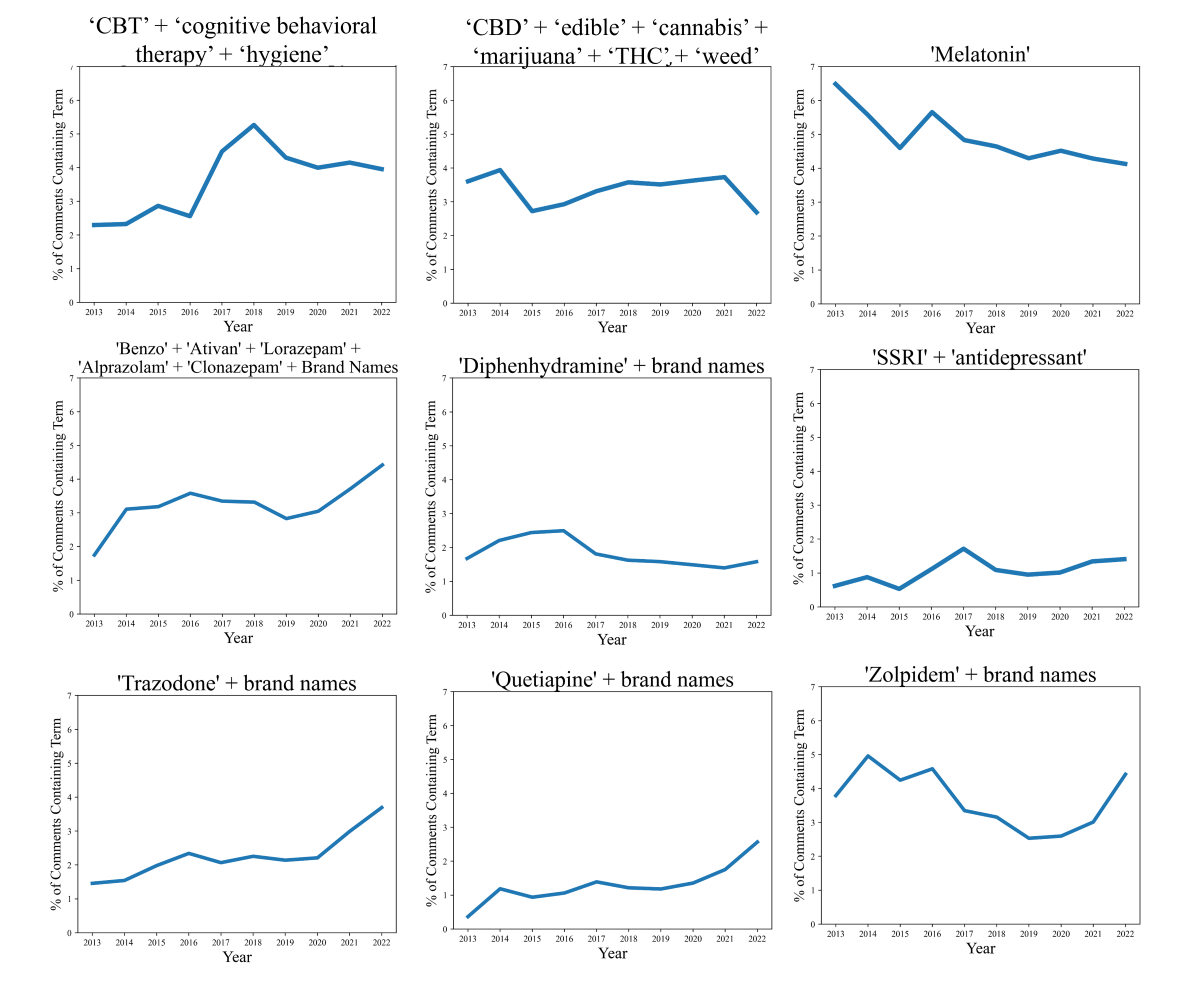
Trends in select treatment and medication terms (percent of comments each year containing specific terms) in the r/insomnia subreddit from 2013 to 2022. As indicated in titles, equivalent or related terms were combined; the numerical percent of comments counts all comments containing one or more of the listed terms. CBT: cognitive behavioral therapy; CBD: cannabidiol; SSRI: selective serotonin reuptake inhibitor; THC: tetrahydrocannabinol.

Unsurprisingly, the most common term in the r/insomnia subreddit was “sleep.” Of the top BOW 1000 terms, we selected 35 terms related to treatment and medication use (Table 1 and Tables S2 and S3 in Multimedia Appendix 1). Melatonin was the most common single treatment term, with 15,005 mentions, followed by terms related to CBT-I, with 13,461 mentions. When time trends were evaluated with RE, terms related to CBT-I spiked in 2017-2018, with a slight decrease and plateau from 2020 onward (Figure 2); the term “hygiene” alone, however, had less variable patterns (Figure S3 in Multimedia Appendix 1). The combined frequency of terms related to antidepressant medications (Table S3 in Multimedia Appendix 1) showed an increase in 2016-2017 and a gradual decline and plateau until a subsequent rise starting in 2020. Terms related to benzodiazepines overall showed a general increase in frequency over time (Figure 2), with a gradual increase from 2013 to 2016, a gradual decrease from 2016 to 2019, and a subsequent increase from 2020 to 2022.

**Table 1:** List of chosen terms related to treatment and medication use, in addition to the number of comments containing at least 1 occurrence of the specific term and the number of comments containing at least 1 occurrence of a combined set of similar terms (data from 2008 to 2022)^a^.

Term	Comments, n
Melatonin	15,005
CBT^b,c^	9018
CBT-I^d^	1512
Hygiene	3846
Ambien	9971
Trazodone^e^	5753
Trazadone	3582
Benzos^f^	3987
Benzo	2289
Benzodiazepine	1158
Weed^g^	3726
Marijuana	1241
Cannabis	1207
CBD^h^	4545
THC^i^	1635
Seroquel	4968
Hygiene	3846
Benadryl	3715
Magnesium	3383
Xanax	3151
Lunesta	2997
Mirtazapine	2902
Antidepressant	2811
SSRI^j^	1469
Zopiclone	2537
Gabapentin^k^	1173
GABA^l^	1133
Valerian	2066
Antihistamine	1638
Klonopin	1514
Zolpidem	1389
Diphenhydramine	1341
Hydroxyzine	1165
Remeron	1047
Ativan	1032
Unisom	812
Quetiapine	940

^a^Combined number of comments are provided over groups of terms that are likely to refer to the same treatment. The number of comments for each unique term may not add up to the combined number of comments because some comments contain multiple terms in a group.

^b^The combined number of comments for the terms CBT, CBT-I, and hygiene together is 13,461.

^c^CBT: cognitive behavioral therapy.

^d^CBT-I: cognitive behavioral therapy for insomnia.

^e^The combined number of comments for the terms trazodone and trazadone together is 9277.

^f^The combined number of comments for the terms benzos, benzo, and benzodiazepine together is 6816.

^g^The combined number of comments for the terms weed, marijuana, cannabis, CBD, and THC together is 10,232.

^h^CBD: cannabidiol.

^i^THC: tetrahydrocannabinol.

^j^SSRI: selective serotonin reuptake inhibitor.

^k^The combined number of comments for the terms gabapentin and GABA together is 2230.

^l^GABA: γ-aminobutyric acid.

### Results of the Treatment and Medication Term Sentiment Analysis

To better understand the emotionality associated with the discussion of treatment within the discussion board, we also conducted a sentiment analysis of the BOW medication and treatment terms. All medication and treatment terms were significantly different from 0 (*P*<.003), except for terms related to “Xanax.” The terms with the most positive sentiment included “hygiene,” “valerian,” “CBT” (referencing CBT-I), “melatonin,” and “CBD” (referencing cannabidiol; Figure 1). The terms with the lowest sentiment (although still positive) included “antidepressant,” “zopiclone,” “Seroquel,” and “Klonopin.”

## Discussion

### Principal Findings

In this analysis of a public internet discussion forum for insomnia, we identified patterns in the frequency of treatment and medication terms over time. Mention of CBT-I, benzodiazepines, trazodone, and other antidepressants increased over time, while nonprescription terms showed varying fluctuations. Our results both align with prior studies and present new findings, particularly for treatments that are not well captured in medical records. Additionally, population-level patterns in sleep health and insomnia may also reflect larger societal changes, given the importance of sociocultural context in sleep behavior [24] as well as the popularity and availability of sleep treatments.

We applied 3 NLP methods. BOW was necessary to identify frequently used terms within the subreddit because it tokenizes each comment and counts words that are used in the greatest number of comments. After BOW was used to identify the most used terms and treatments, RE was a natural choice to determine the number of comments containing the term because RE can identify terms by patterns of letters. As a result, RE can identify not only words that exactly match but also all words that have the same root (including other tenses, plural, closely related words, and some misspelled versions of the word used by commenters). Finally, VADER, a widely used sentiment analysis tool that is specifically trained for social media data, was used to determine in quantifiable terms the degree to which comments containing a term demonstrate positive or negative sentiment.

Individual treatment terms related to melatonin and CBT-I were the most common across time. They were also associated with high positive sentiment. While melatonin frequency showed a slight decline from 2014 to 2022 in the RE analysis, the frequency of terms related to CBT-I spiked after 2016. This rapid increase in CBT-I may be linked to the guidelines published by the American College of Physicians in May 2016, which recommended CBT-I as a frontline treatment for insomnia [12]. However, starting in 2018, there has been a decrease and subsequent plateau in the frequency of CBT-I terms. There is a documented lack of providers trained in behavioral sleep medicine, particularly in CBT-I, where there are not enough CBT-I practitioners to meet patient demand [10,25]. While the reasons for the slight decrease in CBT-I frequency in 2018-2019 are unclear, the plateau from 2020 onward may be due to more limited ability to seek provider care or decreased availability of providers during the COVID-19–related disruptions in the health care system.

The overall increase in the mention of benzodiazepines may indicate a resurgence in benzodiazepine use. Benzodiazepines are a medication of concern because they can become habit-forming. Prior research examining sedative-hypnotic medication prescriptions in the US National Ambulatory Medical Care Survey reported an overall decrease in benzodiazepine prescriptions and an increase in nonbenzodiazepine receptor agonists from 1993 to 2010 [26]. A subsequent analysis of US data from the 1999-2014 National Health and Nutrition Examination Survey supported an increase in benzodiazepine use, which appeared to be driven by medium- and long-term use of the medications [27]. A more recent analysis of benzodiazepine prescriptions from 2018 to 2021 in the United States suggests an uptick during the 2020-2021 COVID-19 pandemic, particularly among women [28], aligning with our findings.

The frequency of terms related to trazodone, an antidepressant with sedative effects that is sometimes prescribed off-label for insomnia, increased steadily until 2016, followed by a plateau from 2017 to 2020, and then rose from 2021 to 2022. A prior analysis of US prescription data from 2011 to 2018 also supports a gradual increase in low-dose (<150 mg) trazodone prescriptions and a concomitant decrease in prescriptions for zolpidem (similar to our findings) [32]. The frequency of terms related to selective serotonin reuptake inhibitors and antidepressants in general differed, with an incline in 2016-2017, followed by a gradual decline and plateau and a slight uptick through 2021-2022. Interestingly, the drop-off in antidepressant term frequency coincides with the peak CBT-I term occurrence. These patterns align with and may be related to the updated clinical guidelines for insomnia treatments published in 2017 by the AASM. This report described an increase in physicians prescribing antidepressants with sedative properties, such as trazodone, as an alternative to benzodiazepines [33]. However, these guidelines also recommended against trazodone and other medications such as tiagabine, diphenhydramine (an antihistamine included in Benadryl), melatonin, and valerian for the treatment of sleep onset or sleep maintenance insomnia [33]. While these guidelines did suggest doxepin, another antidepressant, doxepin was not a common term in the r/insomnia message board. Despite these guidelines and off-label use, trazodone is one of the most commonly prescribed medications for the treatment of insomnia [34].

There were also trends indicating an overall decreased frequency of terms related to OTC medications (such as Benadryl) and marijuana or cannabis-related (non-cannabidiol) terms on r/insomnia. There were opposing trends between cannabidiol (CBD) and cannabis-related non-CBD terms, where CBD frequency had a sharp transient rise after 2016, and cannabis-related non-CBD terms gradually decreased from 2014 onward. This rise in CBD frequency may be due to the relaxation of CBD regulation, a growing interest in the use of cannabinoids for insomnia and sleep [35,36], and interest in the nonpsychoactive properties of CBD in comparison to other cannabinoids. By 2016, a majority of US states had legalized medical cannabis or CBD [37], and in 2018, hemp-derived CBD was removed as a Schedule I substance in accordance with the 2018 Agriculture Improvement Act [38], increasing its availability to US consumers; however, there was a rapid decline in mentions of CBD in 2022. Although the extent of cannabis use for insomnia treatment and directionality is unclear, prior work suggests a high prevalence of insomnia symptoms and sleep disturbances among people who use cannabis, with 97% increased odds of insomnia among participants reporting daily cannabis use [39]. As cannabis products become more widely available, these findings support the need for greater investigation of the prevalence, possible benefits, and possible drawbacks of cannabis use for insomnia symptoms.

The use of social data may provide an alternative method to capture treatment trends that may be underreported by conventional methods. The 2017 AASM report speculated that the true use of sleep medications for insomnia is higher than reported [33]. Likewise, a prior analysis of US 1999-2010 National Health and Nutrition Examination Survey data found that of those participants who reported using a sleep aid medication, 58% did not provide information for prescription medication, suggesting widespread use of OTC or alternative treatments [40]. Our findings also suggest a high prevalence of the mention of nonprescription sleep aids on r/insomnia and suggest that digital discussion boards may provide an alternative means of investigating these patterns. Additionally, sleep is a physiological process that can be influenced by sociocultural factors, as it is nested within a socioecological context of the individual, social factors, and society [41-43]. Because of this, social and cultural phenomena, such as societal change and upheaval, can impact sleep and insomnia. For example, measures of self-reported sleep and mood worsened during the week of the US 2020 election compared to a baseline measure a few weeks prior in both US and non-US participants [44]. Our results also suggest changepoints in treatment terms around 2016 and 2020, which may be related to political election cycles or the COVID-19 pandemic. Future research could more explicitly investigate the relationships between sleep health and social and societal factors using r/insomnia and other digital communities.

In addition to CBT-I, r/insomnia comments reflected positive sentiment toward “natural” or herbal supplements and therapies, such as melatonin, valerian root, and cannabidiol. Counter to our expectations that the term would have a negative connotation and sentiment, the term “hygiene,” referencing the sleep hygiene component of CBT-I, was the term with the highest positive sentiment. This may reflect that the commenters were not bothered by the term or that this CBT-I component was successful in improving insomnia symptoms. The median sentiment for comments containing each term was positive, which may suggest that the data from this subreddit may be more positive than the median sentiment from the dataset VADER was trained on.

### Strengths and Limitations

Our analysis has multiple strengths and limitations, which should be considered in interpreting results. Because the r/insomnia subreddit is accessible to people all over the world, the term trends may reflect an international sample of posts. Yet, it is not representative of a population with defined characteristics, as individuals who participated in the subreddit are self-selected. Our analysis was at the level of comments in response to original posts, treating each comment as independent. Therefore, the analysis may be biased by the overrepresentation of large threads focusing on specific topics. While sentiment analysis attempts to identify the general emotionality of text, there are some limitations; it is not able to capture sarcasm, and because the text is considered as a whole, text with both positive and negative comments will be treated as a composite rather than as an individual component. While we sought to include alternative or brand names for medications in trend measurement, treatments with many options or medication names (such as that for selective serotonin reuptake inhibitors) may exhibit residual underrepresentation or bias.

### Conclusions

The use of language related to CBT-I and medications such as benzodiazepines and trazodone and other antidepressants have fluctuated over time on the r/insomnia Reddit platform. Some of these trends, such as the rise in CBT-I in 2017, may reflect clinical treatment guidelines, while others align with nationwide prescription trends. Trends of treatment-related terms, such as the changepoints around 2016 and 2020, may also reflect larger societal events, such as the 2020 COVID-19 pandemic. The data also suggest that r/insomnia commenters mention treatment terms related to OTC and alternative therapies, such as melatonin and cannabis. The representation of OTC and alternative treatments that are not captured by prescription activity is an especially important aspect of this dataset, as the use of nonprescription sleep aids is difficult to track; such information may be useful for sleep health practitioners. The r/insomnia dataset or other alternative data sources may be a valuable addition to prescription records for future studies seeking to capture nonprescription sleep aid use. The prevalence of these terms in the data suggests that future studies of insomnia should consider collecting information on OTC treatment use, cannabis use, and other alternative therapies that people may seek when experiencing insomnia symptoms. Data from digital communities such as r/insomnia may also provide a deeper understanding of how social and societal factors shape sleep health globally.

## Appendix

Multimedia Appendix 1 Additional tables and figures of treatment terms and trends from the r/insomnia subreddit.

## Abbreviations

AASM: American Academy of Sleep Medicine
BOW: bag of words
CBD: cannabidiol
CBT-I: cognitive behavioral therapy for insomnia
NLP: natural language processing
OTC: over-the-counter
RE: regular expression
VADER: Valence Aware Dictionary and Sentiment Reasoner

## Data Availability

The r/insomnia Reddit data analyzed in this study are publicly available in Academic Torrents [45-47].
