# Supplementary material for: Applying Natural Language Processing Techniques to Map Trends in Insomnia Treatment Terms on the r/Insomnia Subreddit: Infodemiology Study
Source: J Med Internet Res. 2025 Jan 9;27:e58902. doi: 10.2196/58902 (PMC11757973; doi:10.2196/58902)
Supplement: Multimedia Appendix 1 [file jmir_v27i1e58902_app1.docx]

**Table S1.** Number of comments and unique commenters in the r/insomnia Reddit discussion board by year.

| **Year** | **Number of Comments** | **Number of Users With at Least One Comment** | **Number of Comments per Unique Commenter** |
| --- | --- | --- | --- |
| 2011 | 306 | 155 | 1.97 |
| 2012 | 1,832 | 581 | 3.15 |
| 2013 | 2,744 | 841 | 3.26 |
| 2014 | 4,213 | 1,237 | 3.41 |
| 2015 | 5,653 | 1,538 | 3.68 |
| 2016 | 7,815 | 1,952 | 4.00 |
| 2017 | 11,651 | 2,750 | 4.24 |
| 2018 | 26,333 | 4,875 | 5.40 |
| 2019 | 47,275 | 9,194 | 5.14 |
| 2020 | 68,833 | 12,914 | 5.33 |
| 2021 | 76,767 | 12,584 | 6.10 |
| 2022 | 86,623 | 14,866 | 5.83 |

**Table S2**. The top 1,000 most common words and frequency counts in r/insomnia returned from the BOW model.

| **Word** | **Count** |
| --- | --- |
| sleep | 120327 |
| like | 57740 |
| insomnia | 50330 |
| night | 47075 |
| time | 46498 |
| help | 43200 |
| day | 41708 |
| work | 39605 |
| hour | 36963 |
| try | 35564 |
| know | 32890 |
| feel | 32458 |
| thing | 31802 |
| good | 30594 |
| think | 27991 |
| anxiety | 26605 |
| bed | 26234 |
| sleeping | 26125 |
| asleep | 26050 |
| going | 22816 |
| doctor | 22507 |
| people | 22126 |
| year | 22121 |
| need | 21525 |
| better | 21410 |
| tried | 18726 |
| taking | 18366 |
| long | 18139 |
| fall | 18118 |
| wake | 18016 |
| way | 17988 |
| week | 17923 |
| lot | 17782 |
| getting | 17172 |
| mg | 16765 |
| issue | 16544 |
| problem | 16425 |
| got | 16129 |
| med | 15551 |
| life | 15536 |
| bad | 15503 |
| use | 15421 |
| body | 15059 |
| melatonin | 15005 |
| want | 14938 |
| effect | 14903 |
| deleted | 14897 |
| maybe | 14794 |
| right | 14663 |
| month | 14319 |
| sure | 14062 |
| hope | 13862 |
| medication | 13757 |
| best | 13724 |
| trying | 13584 |
| actually | 13488 |
| thank | 13485 |
| thought | 12746 |
| awake | 12662 |
| cause | 12309 |
| able | 12248 |
| yes | 12128 |
| start | 12060 |
| usually | 11819 |
| sound | 11693 |
| brain | 11584 |
| thanks | 11497 |
| yeah | 11201 |
| tired | 11167 |
| mind | 11131 |
| removed | 11033 |
| started | 11032 |
| make | 10710 |
| drug | 10657 |
| probably | 10637 |
| pill | 10558 |
| helped | 10455 |
| feeling | 10326 |
| stay | 10099 |
| ambien | 9971 |
| definitely | 9901 |
| morning | 9830 |
| pretty | 9683 |
| hard | 9645 |
| little | 9563 |
| took | 9425 |
| different | 9152 |
| luck | 9033 |
| stress | 9024 |
| cbt | 9018 |
| worse | 9007 |
| look | 8855 |
| worked | 8829 |
| experience | 8791 |
| said | 8731 |
| working | 8672 |
| dose | 8645 |
| come | 8443 |
| slept | 8442 |
| sorry | 8422 |
| stop | 8386 |
| point | 8374 |
| normal | 8290 |
| bit | 8278 |
| great | 8260 |
| read | 8142 |
| lol | 8101 |
| light | 8060 |
| mean | 7914 |
| went | 7763 |
| let | 7727 |
| post | 7597 |
| term | 7164 |
| http | 7117 |
| kind | 7028 |
| tell | 7008 |
| minute | 6865 |
| away | 6862 |
| short | 6840 |
| advice | 6794 |
| ago | 6788 |
| exercise | 6757 |
| instead | 6558 |
| reason | 6518 |
| waking | 6508 |
| stuff | 6506 |
| high | 6387 |
| health | 6309 |
| prescribed | 6289 |
| couple | 6197 |
| thinking | 6135 |
| depression | 6114 |
| shit | 6111 |
| ask | 6098 |
| schedule | 6080 |
| therapy | 6080 |
| doc | 6046 |
| symptom | 6043 |
| pm | 6011 |
| idea | 5995 |
| falling | 5988 |
| end | 5984 |
| case | 5969 |
| talk | 5949 |
| unfortunately | 5842 |
| trazodone | 5753 |
| question | 5662 |
| study | 5652 |
| half | 5627 |
| past | 5539 |
| new | 5522 |
| rest | 5503 |
| eye | 5437 |
| later | 5416 |
| hear | 5403 |
| remember | 5390 |
| wish | 5383 |
| soon | 5381 |
| early | 5375 |
| hey | 5355 |
| im | 5355 |
| low | 5345 |
| felt | 5268 |
| worry | 5241 |
| understand | 5214 |
| change | 5209 |
| head | 5148 |
| dream | 5118 |
| recommend | 5106 |
| suck | 5080 |
| friend | 5044 |
| solution | 5041 |
| check | 5008 |
| fine | 4999 |
| far | 4990 |
| mental | 4973 |
| seroquel | 4968 |
| meditation | 4968 |
| similar | 4894 |
| sleepy | 4873 |
| happens | 4873 |
| possible | 4855 |
| cycle | 4828 |
| get | 4824 |
| completely | 4799 |
| person | 4797 |
| eventually | 4771 |
| especially | 4755 |
| oh | 4741 |
| chronic | 4725 |
| likely | 4705 |
| anxious | 4699 |
| effective | 4667 |
| book | 4631 |
| medical | 4619 |
| room | 4614 |
| guess | 4613 |
| stopped | 4612 |
| phone | 4605 |
| worth | 4587 |
| care | 4565 |
| drink | 4562 |
| literally | 4556 |
| cbd | 4545 |
| com | 4528 |
| worst | 4514 |
| taken | 4458 |
| prescription | 4453 |
| longer | 4449 |
| disorder | 4442 |
| job | 4421 |
| amp | 4417 |
| tolerance | 4397 |
| take | 4390 |
| weird | 4365 |
| pain | 4335 |
| free | 4332 |
| helpful | 4329 |
| told | 4309 |
| turn | 4289 |
| man | 4244 |
| finally | 4237 |
| heard | 4219 |
| reading | 4211 |
| supplement | 4200 |
| ok | 4191 |
| believe | 4182 |
| dont | 4166 |
| psychiatrist | 4165 |
| magnesium | 4165 |
| edit | 4139 |
| go | 4121 |
| recommended | 4119 |
| www | 4093 |
| live | 4088 |
| level | 4074 |
| training | 4056 |
| state | 4055 |
| exactly | 4055 |
| real | 4052 |
| relax | 4031 |
| love | 4004 |
| insomniac | 4002 |
| caffeine | 3989 |
| benzos | 3987 |
| nap | 3976 |
| super | 3973 |
| honestly | 3972 |
| anymore | 3930 |
| matter | 3917 |
| calm | 3882 |
| watch | 3879 |
| happen | 3876 |
| tonight | 3867 |
| hygiene | 3846 |
| important | 3846 |
| common | 3843 |
| situation | 3841 |
| late | 3839 |
| gave | 3833 |
| making | 3827 |
| called | 3821 |
| eat | 3802 |
| looking | 3778 |
| lack | 3768 |
| therapist | 3750 |
| place | 3749 |
| heart | 3747 |
| weed | 3726 |
| fix | 3716 |
| benadryl | 3715 |
| course | 3705 |
| control | 3697 |
| big | 3689 |
| trouble | 3687 |
| absolutely | 3686 |
| noise | 3675 |
| message | 3647 |
| fact | 3629 |
| today | 3622 |
| aid | 3619 |
| basically | 3608 |
| youtube | 3596 |
| second | 3591 |
| trazadone | 3582 |
| school | 3562 |
| recently | 3560 |
| severe | 3537 |
| type | 3530 |
| deep | 3515 |
| concern | 3509 |
| breathing | 3482 |
| natural | 3478 |
| home | 3475 |
| withdrawal | 3473 |
| happened | 3472 |
| staying | 3463 |
| understanding | 3451 |
| method | 3391 |
| set | 3380 |
| rhythm | 3371 |
| alcohol | 3369 |
| treatment | 3362 |
| period | 3361 |
| bedtime | 3355 |
| glad | 3344 |
| deal | 3343 |
| subreddit | 3329 |
| option | 3325 |
| hopefully | 3293 |
| habit | 3288 |
| okay | 3288 |
| currently | 3286 |
| sort | 3283 |
| result | 3282 |
| woke | 3281 |
| circadian | 3275 |
| video | 3275 |
| routine | 3272 |
| action | 3236 |
| quality | 3207 |
| caused | 3199 |
| gonna | 3196 |
| weight | 3175 |
| hell | 3175 |
| consistent | 3170 |
| needed | 3169 |
| crazy | 3167 |
| hr | 3164 |
| xanax | 3151 |
| old | 3143 |
| contact | 3132 |
| avoid | 3126 |
| condition | 3121 |
| gt | 3121 |
| lay | 3110 |
| listen | 3104 |
| self | 3091 |
| small | 3090 |
| panic | 3090 |
| saying | 3089 |
| easy | 3086 |
| apnea | 3073 |
| medicine | 3065 |
| personally | 3062 |
| TRUE | 3056 |
| hit | 3048 |
| cold | 3048 |
| fear | 3046 |
| physical | 3045 |
| comment | 3043 |
| break | 3036 |
| hate | 3035 |
| fuck | 3024 |
| answer | 2999 |
| lunesta | 2997 |
| causing | 2993 |
| focus | 2988 |
| easier | 2985 |
| family | 2985 |
| knock | 2968 |
| tea | 2962 |
| suggest | 2956 |
| blood | 2955 |
| root | 2954 |
| require | 2948 |
| story | 2936 |
| wrong | 2931 |
| sense | 2928 |
| close | 2927 |
| energy | 2913 |
| treat | 2913 |
| happy | 2902 |
| mirtazapine | 2902 |
| exhausted | 2901 |
| nice | 2895 |
| non | 2895 |
| seeing | 2876 |
| bot | 2876 |
| middle | 2874 |
| horrible | 2872 |
| daily | 2866 |
| starting | 2863 |
| regular | 2858 |
| dos | 2854 |
| talking | 2851 |
| text | 2850 |
| min | 2840 |
| dosage | 2836 |
| related | 2834 |
| totally | 2824 |
| nightmare | 2816 |
| antidepressant | 2811 |
| negative | 2804 |
| eating | 2792 |
| struggle | 2786 |
| run | 2775 |
| automatically | 2768 |
| agree | 2754 |
| attack | 2737 |
| gone | 2728 |
| test | 2721 |
| reddit | 2716 |
| anti | 2710 |
| difficult | 2698 |
| terrible | 2683 |
| consider | 2680 |
| compose | 2678 |
| coffee | 2677 |
| tv | 2677 |
| underlying | 2675 |
| straight | 2668 |
| affect | 2659 |
| function | 2656 |
| safe | 2651 |
| dr | 2650 |
| clock | 2642 |
| extremely | 2638 |
| field | 2634 |
| interesting | 2630 |
| food | 2627 |
| pressure | 2619 |
| difference | 2615 |
| keeping | 2600 |
| helping | 2594 |
| fucking | 2583 |
| one | 2572 |
| earlier | 2571 |
| healthy | 2564 |
| wonder | 2555 |
| deprivation | 2542 |
| performed | 2542 |
| zopiclone | 2537 |
| research | 2527 |
| water | 2525 |
| character | 2524 |
| increase | 2521 |
| patience | 2517 |
| given | 2513 |
| moderator | 2506 |
| submission | 2502 |
| give | 2500 |
| seen | 2488 |
| resubmit | 2488 |
| strong | 2469 |
| cognitive | 2465 |
| prescribe | 2457 |
| normally | 2444 |
| vitamin | 2436 |
| wanted | 2434 |
| substance | 2421 |
| world | 2404 |
| blue | 2397 |
| came | 2383 |
| specialist | 2366 |
| pattern | 2363 |
| shift | 2363 |
| restriction | 2360 |
| play | 2359 |
| form | 2358 |
| music | 2349 |
| editing | 2349 |
| online | 2348 |
| relaxing | 2347 |
| screen | 2343 |
| general | 2334 |
| walk | 2334 |
| drinking | 2332 |
| comply | 2332 |
| gotten | 2326 |
| automoderator | 2320 |
| reapprove | 2320 |
| keep | 2310 |
| blanket | 2297 |
| quickly | 2292 |
| benzo | 2289 |
| dark | 2273 |
| kinda | 2269 |
| awful | 2267 |
| chance | 2260 |
| certain | 2259 |
| coming | 2257 |
| white | 2244 |
| alarm | 2244 |
| figure | 2242 |
| add | 2223 |
| suffer | 2214 |
| dealing | 2194 |
| seriously | 2193 |
| happening | 2180 |
| hot | 2171 |
| lower | 2160 |
| process | 2157 |
| haha | 2156 |
| op | 2155 |
| word | 2153 |
| quit | 2152 |
| slowly | 2151 |
| app | 2146 |
| guy | 2141 |
| tip | 2141 |
| worried | 2136 |
| diet | 2133 |
| build | 2133 |
| moment | 2130 |
| lead | 2129 |
| groggy | 2128 |
| positive | 2126 |
| manage | 2126 |
| oil | 2126 |
| scared | 2124 |
| exact | 2122 |
| noticed | 2120 |
| addictive | 2119 |
| pas | 2117 |
| cure | 2106 |
| muscle | 2088 |
| improve | 2088 |
| experienced | 2085 |
| idk | 2083 |
| age | 2079 |
| response | 2079 |
| suggestion | 2078 |
| single | 2078 |
| plus | 2067 |
| valerian | 2066 |
| benefit | 2053 |
| higher | 2049 |
| counter | 2045 |
| easily | 2043 |
| die | 2032 |
| drive | 2027 |
| program | 2019 |
| suffering | 2019 |
| open | 2018 |
| mentioned | 2016 |
| memory | 2014 |
| experiencing | 2013 |
| cut | 1999 |
| evening | 1995 |
| harder | 1991 |
| plan | 1987 |
| risk | 1984 |
| say | 1978 |
| entire | 1975 |
| hand | 1966 |
| onset | 1964 |
| ive | 1962 |
| example | 1962 |
| giving | 1958 |
| watching | 1951 |
| fast | 1950 |
| supposed | 1949 |
| huge | 1948 |
| game | 1944 |
| naturally | 1943 |
| drowsy | 1937 |
| parent | 1928 |
| stressed | 1927 |
| appreciate | 1927 |
| ended | 1924 |
| realize | 1923 |
| tend | 1922 |
| listening | 1920 |
| tomorrow | 1919 |
| damn | 1915 |
| otc | 1913 |
| leg | 1907 |
| living | 1903 |
| wait | 1894 |
| hoping | 1892 |
| kid | 1887 |
| outside | 1887 |
| physically | 1884 |
| sub | 1884 |
| god | 1883 |
| laying | 1882 |
| including | 1881 |
| struggling | 1871 |
| total | 1867 |
| house | 1867 |
| learn | 1863 |
| aware | 1863 |
| based | 1858 |
| till | 1842 |
| key | 1841 |
| smoke | 1835 |
| leave | 1832 |
| rem | 1830 |
| heavy | 1829 |
| force | 1822 |
| generally | 1821 |
| mood | 1820 |
| wow | 1818 |
| technique | 1813 |
| diagnosed | 1812 |
| notice | 1812 |
| sun | 1811 |
| asking | 1811 |
| tho | 1810 |
| adhd | 1799 |
| sick | 1797 |
| frustrating | 1795 |
| disease | 1794 |
| deprived | 1791 |
| appointment | 1790 |
| cool | 1789 |
| everyday | 1788 |
| thats | 1787 |
| lucky | 1784 |
| spend | 1781 |
| rule | 1780 |
| lost | 1778 |
| info | 1777 |
| buy | 1773 |
| relief | 1765 |
| stick | 1760 |
| afraid | 1758 |
| constantly | 1758 |
| imagine | 1756 |
| obviously | 1752 |
| shot | 1751 |
| multiple | 1742 |
| fell | 1738 |
| highly | 1731 |
| headache | 1729 |
| kept | 1729 |
| hormone | 1724 |
| telling | 1722 |
| racing | 1720 |
| left | 1719 |
| reply | 1712 |
| fan | 1704 |
| wide | 1704 |
| amazing | 1704 |
| finding | 1702 |
| shut | 1696 |
| window | 1691 |
| hallucination | 1685 |
| gain | 1685 |
| stomach | 1684 |
| yoga | 1682 |
| standard | 1681 |
| activity | 1675 |
| saw | 1667 |
| restless | 1667 |
| step | 1666 |
| specific | 1664 |
| order | 1658 |
| hurt | 1657 |
| careful | 1656 |
| practice | 1654 |
| professional | 1652 |
| comfortable | 1644 |
| hospital | 1643 |
| trick | 1643 |
| sleeper | 1643 |
| antihistamine | 1638 |
| number | 1635 |
| thc | 1635 |
| list | 1635 |
| mention | 1632 |
| dude | 1630 |
| fully | 1627 |
| fight | 1626 |
| knowing | 1624 |
| ton | 1624 |
| mess | 1619 |
| immediately | 1617 |
| actual | 1614 |
| simply | 1614 |
| lose | 1611 |
| trust | 1605 |
| twice | 1601 |
| accept | 1599 |
| bc | 1599 |
| follow | 1598 |
| changed | 1596 |
| sleepless | 1596 |
| tablet | 1593 |
| patient | 1593 |
| dangerous | 1592 |
| fun | 1589 |
| reset | 1587 |
| mom | 1587 |
| yea | 1585 |
| hi | 1584 |
| depressed | 1581 |
| lie | 1578 |
| following | 1577 |
| active | 1574 |
| ability | 1572 |
| track | 1570 |
| factor | 1570 |
| extra | 1569 |
| relate | 1569 |
| google | 1565 |
| scary | 1564 |
| relaxation | 1563 |
| turning | 1562 |
| episode | 1562 |
| barely | 1558 |
| impossible | 1558 |
| known | 1555 |
| unable | 1552 |
| smoking | 1548 |
| insurance | 1546 |
| switch | 1543 |
| face | 1541 |
| rested | 1534 |
| yep | 1534 |
| ready | 1533 |
| pillow | 1525 |
| learned | 1520 |
| phase | 1520 |
| truly | 1520 |
| specifically | 1515 |
| klonopin | 1514 |
| history | 1512 |
| cbti | 1512 |
| weekend | 1509 |
| worrying | 1508 |
| lying | 1504 |
| success | 1501 |
| tough | 1493 |
| rare | 1492 |
| support | 1491 |
| permanent | 1489 |
| certainly | 1488 |
| sugar | 1485 |
| baby | 1483 |
| zero | 1482 |
| bedroom | 1480 |
| poor | 1480 |
| slow | 1478 |
| usual | 1478 |
| regularly | 1477 |
| addiction | 1477 |
| class | 1473 |
| wear | 1472 |
| possibly | 1472 |
| warm | 1470 |
| ssri | 1469 |
| brand | 1469 |
| continue | 1469 |
| amazon | 1468 |
| lately | 1463 |
| mentally | 1462 |
| proper | 1462 |
| article | 1454 |
| average | 1453 |
| syndrome | 1446 |
| rough | 1444 |
| simple | 1441 |
| enjoy | 1440 |
| relaxed | 1439 |
| maintenance | 1438 |
| release | 1436 |
| clear | 1435 |
| knew | 1434 |
| bipolar | 1434 |
| write | 1433 |
| depends | 1431 |
| covid | 1429 |
| ear | 1428 |
| rate | 1428 |
| consistently | 1424 |
| reduce | 1422 |
| source | 1420 |
| weighted | 1418 |
| typically | 1414 |
| personal | 1413 |
| act | 1410 |
| main | 1409 |
| putting | 1406 |
| available | 1406 |
| shower | 1404 |
| behavioral | 1401 |
| kick | 1400 |
| link | 1400 |
| kill | 1391 |
| zolpidem | 1389 |
| decided | 1383 |
| developed | 1382 |
| primary | 1367 |
| share | 1365 |
| major | 1358 |
| random | 1354 |
| impact | 1354 |
| extreme | 1354 |
| perfect | 1353 |
| opposite | 1351 |
| ptsd | 1350 |
| running | 1347 |
| decent | 1346 |
| sharing | 1345 |
| stressful | 1343 |
| diphenhydramine | 1341 |
| row | 1336 |
| meant | 1336 |
| wanna | 1336 |
| dependent | 1334 |
| miss | 1334 |
| explain | 1333 |
| money | 1330 |
| information | 1328 |
| org | 1326 |
| address | 1325 |
| human | 1325 |
| apparently | 1317 |
| drop | 1317 |
| hang | 1316 |
| choice | 1311 |
| psych | 1307 |
| sit | 1300 |
| opinion | 1296 |
| net | 1294 |
| quick | 1293 |
| illness | 1293 |
| bring | 1291 |
| mild | 1286 |
| combination | 1283 |
| forget | 1282 |
| cortisol | 1281 |
| vivid | 1279 |
| husband | 1275 |
| nearly | 1274 |
| decade | 1268 |
| taper | 1263 |
| pay | 1261 |
| somewhat | 1260 |
| stuck | 1254 |
| playing | 1254 |
| stage | 1246 |
| looked | 1245 |
| boat | 1241 |
| marijuana | 1241 |
| annoying | 1240 |
| yesterday | 1236 |
| walking | 1236 |
| beat | 1233 |
| reaction | 1231 |
| line | 1230 |
| depending | 1228 |
| improvement | 1228 |
| gotta | 1227 |
| young | 1227 |
| properly | 1223 |
| breath | 1220 |
| mouth | 1220 |
| ill | 1219 |
| asked | 1217 |
| allow | 1216 |
| couch | 1211 |
| nervous | 1211 |
| addicted | 1210 |
| checked | 1208 |
| cannabis | 1207 |
| treating | 1206 |
| sedative | 1204 |
| college | 1201 |
| trigger | 1200 |
| considered | 1200 |
| speak | 1200 |
| er | 1199 |
| managed | 1198 |
| fucked | 1197 |
| product | 1197 |
| occasionally | 1189 |
| near | 1188 |
| approach | 1187 |
| bout | 1186 |
| note | 1185 |
| begin | 1185 |
| waiting | 1183 |
| stupid | 1182 |
| particularly | 1179 |
| hold | 1177 |
| ya | 1177 |
| gabapentin | 1173 |
| adult | 1172 |
| changing | 1172 |
| combo | 1170 |
| bunch | 1169 |
| child | 1165 |
| hydroxyzine | 1165 |
| beginning | 1164 |
| napping | 1164 |
| nightly | 1159 |
| benzodiazepine | 1158 |
| mask | 1157 |
| constant | 1156 |
| wind | 1153 |
| gp | 1153 |
| moving | 1153 |
| podcast | 1150 |
| desperate | 1149 |
| dead | 1148 |
| awesome | 1146 |
| damage | 1143 |
| glass | 1141 |
| improved | 1140 |
| turned | 1139 |
| gaba | 1133 |
| lifestyle | 1130 |
| machine | 1130 |
| suddenly | 1129 |
| afternoon | 1128 |
| expensive | 1127 |
| useful | 1127 |
| spent | 1126 |
| regardless | 1125 |
| incredibly | 1119 |
| temperature | 1118 |
| zombie | 1118 |
| excessive | 1115 |
| develop | 1115 |
| jerk | 1114 |
| forever | 1113 |
| daytime | 1113 |
| despite | 1113 |
| hangover | 1110 |
| fairly | 1108 |
| overall | 1106 |
| internet | 1106 |
| current | 1104 |
| forward | 1103 |
| curious | 1102 |
| complete | 1101 |
| country | 1101 |
| insomniasos | 1095 |
| intense | 1093 |
| doubt | 1093 |
| delayed | 1092 |
| mix | 1087 |
| death | 1086 |
| as | 1084 |
| realized | 1083 |
| depressant | 1082 |
| fatal | 1081 |
| push | 1080 |
| reach | 1079 |
| loss | 1076 |
| didnt | 1075 |
| catch | 1071 |
| driving | 1069 |
| interested | 1068 |
| limit | 1068 |
| honest | 1065 |
| bright | 1065 |
| rarely | 1064 |
| quiet | 1062 |
| edible | 1061 |
| area | 1059 |
| attention | 1058 |
| adjust | 1058 |
| repeat | 1055 |
| psychological | 1055 |
| bought | 1054 |
| alternative | 1052 |
| shitty | 1052 |
| trauma | 1051 |
| faster | 1050 |
| evidence | 1050 |
| wondering | 1049 |
| social | 1048 |
| air | 1048 |
| solid | 1047 |
| remeron | 1047 |
| fatigue | 1045 |
| insane | 1043 |
| rebound | 1042 |
| strange | 1041 |
| calming | 1041 |
| broken | 1040 |
| crap | 1040 |
| suggested | 1040 |
| increased | 1039 |
| expect | 1037 |
| handle | 1036 |
| mode | 1032 |
| ativan | 1032 |
| turkey | 1031 |
| diagnosis | 1029 |
| awhile | 1023 |
| letting | 1022 |
| dreaming | 1019 |
| paralysis | 1017 |
| closed | 1014 |
| psychologist | 1013 |
| offer | 1011 |
| sad | 1008 |
| boring | 1005 |
| plenty | 1002 |
| movie | 1001 |
| unisom | 999 |
| legal | 998 |
| sign | 997 |
| messed | 996 |
| event | 996 |


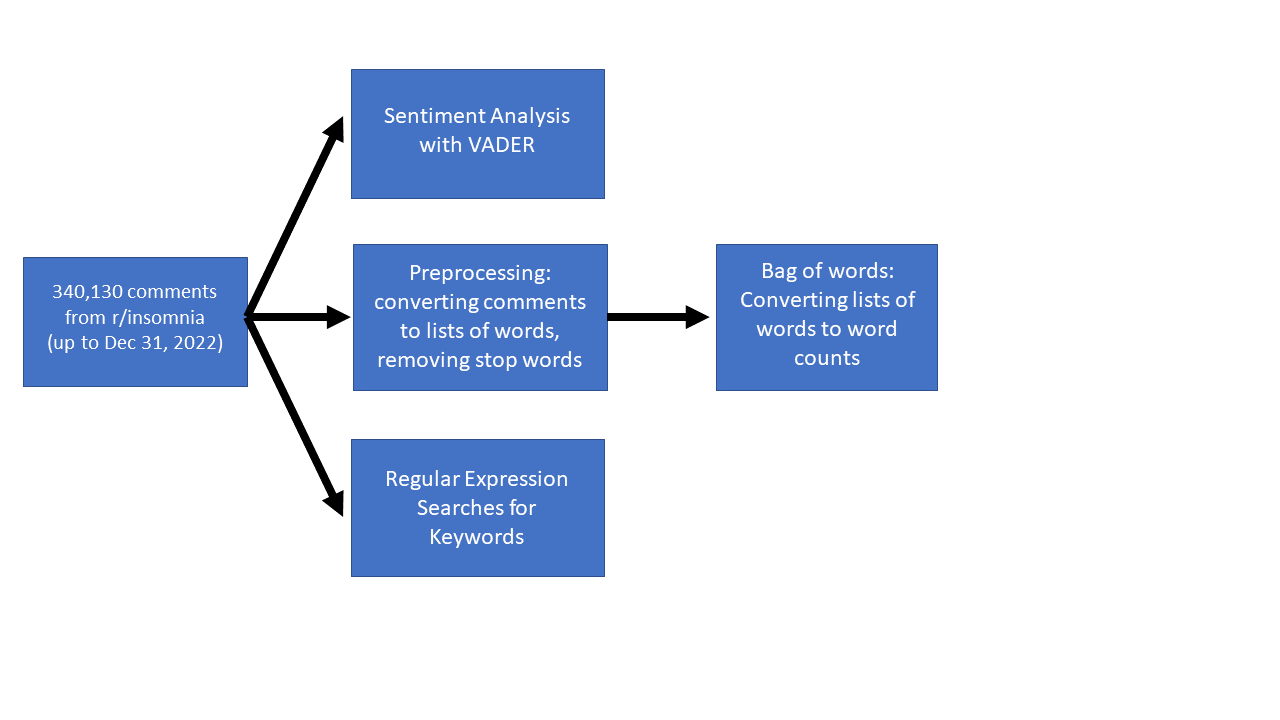


**Figure S1***.* Flowchart depicting natural language processing methods for the r/insomnia Reddit analysis.


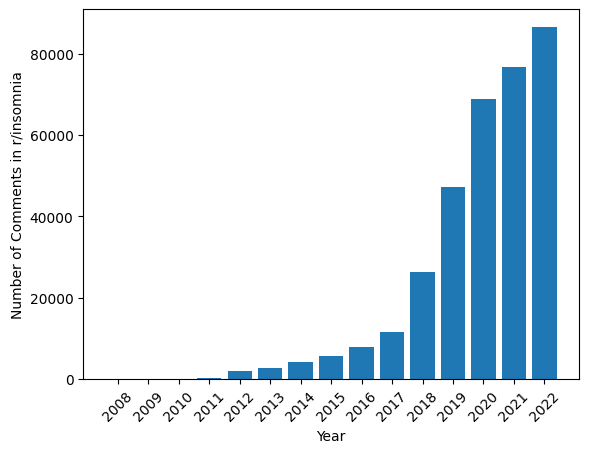


**Figure S2***.* Number of comments in r/insomnia Reddit discussion board by year.

**Table S3***.* Generic names, brand names, and common abbreviations and misspellings for medications analyzed in this study. These terms were used to generate overtime trend plots using RE keyword searches (data from 2013-2022).

| **Generic Name** | **Brand Names + Common Misspellings and Abbreviations** | **# of Comments Containing at Least One Term** |
| --- | --- | --- |
| Zolpidem | Ambien, Ambien CR, Edluar, Intermezzo, Zolpimist | 11,256 |
| Trazodone | Desyrl, Desyrel Dividose Oleptro Trazadone D, Donaren, Molipaxin, Trazorel, Trittico, trazadone | 9,262 |
| Diphenhydramine | Banophen, Benadryl Allergy, Benadryl Allergy Extra Strength, Children's Benadryl Allergy, Nytol QuickCaps, Simply Sleep, Sominex Maximum Strength, Sominex Original, Unisom SleepGels, Unisom SleepMelts, Unisom SleepMinis, Vicks ZzzQuil, Dimedrol, Daedalon, Benadryl, Unisom | 5,327 |
| Quetiapine | Seroquel, Seroquel XR, Atrolak, Biquelle, Sondate, Zaluron | 5,739 |
| Alprazolam | Niravam, Xanax, Xanax XR | 3,310 |
| Eszopiclone | Lunesta | 3,130 |
| Gabapentin | Neurontin, Gralise, Gabarone, FusePaq Fanatrex, Horizant, Gaba | 2,544 |
| Clonazepam | Klonopin, Klonopin Wafers, Rivotril, Clonapam | 2,406 |
| Hydroxyzine | Atarax, Vistaril, Equipose, Masmoran, Paxistil, Alamon, Aterax, Durrax, Tran-Q, Orgatrax, Quiess, Tranquizine | 1,395 |
| Mirtazapine | Remeron, Remeron SolTab, Remeron RD, Mirtax | 3,816 |
| Lorazepam | Loreev XR, Ativan, Lorazepam Intensol | 1,462 |


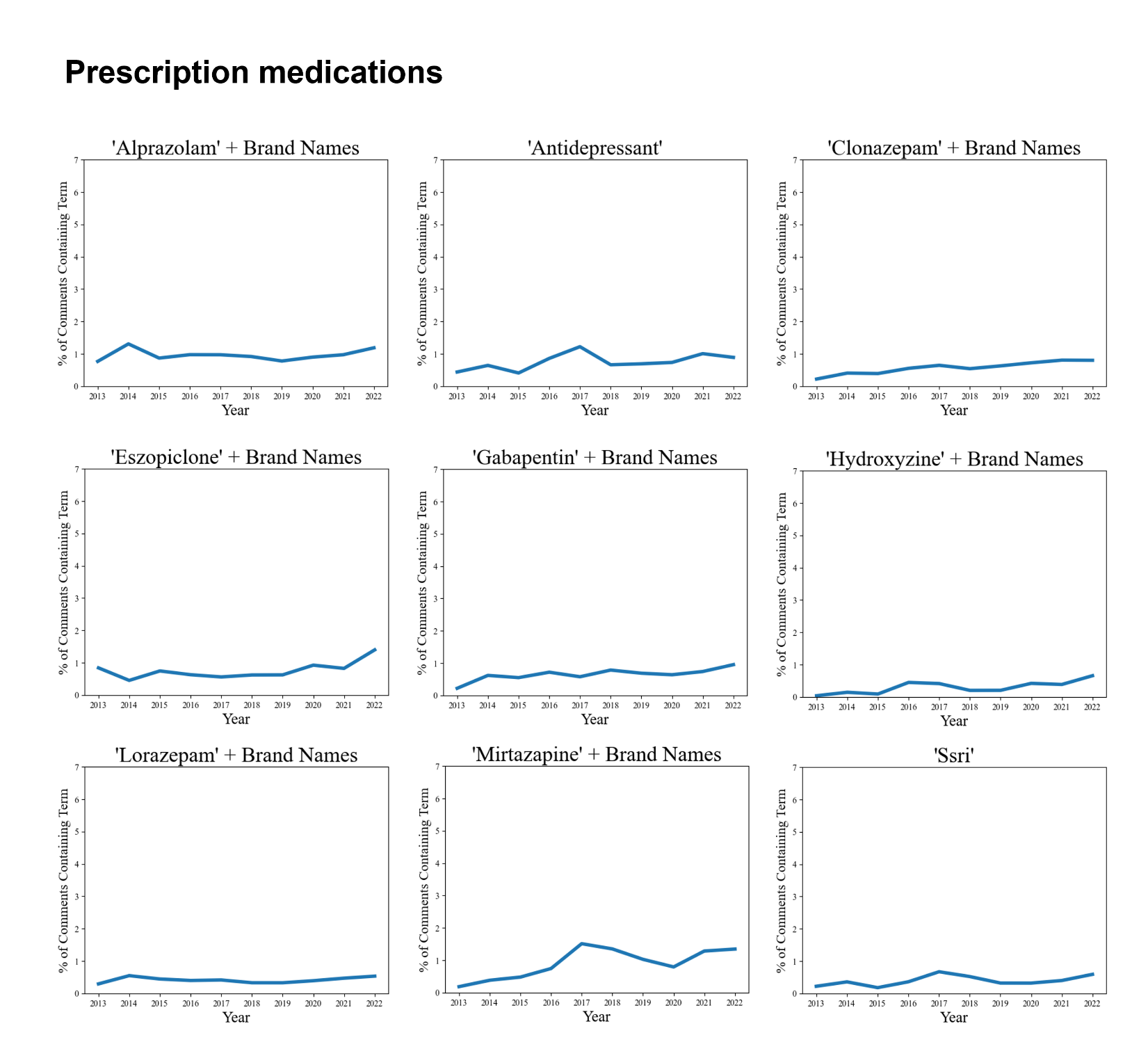


**
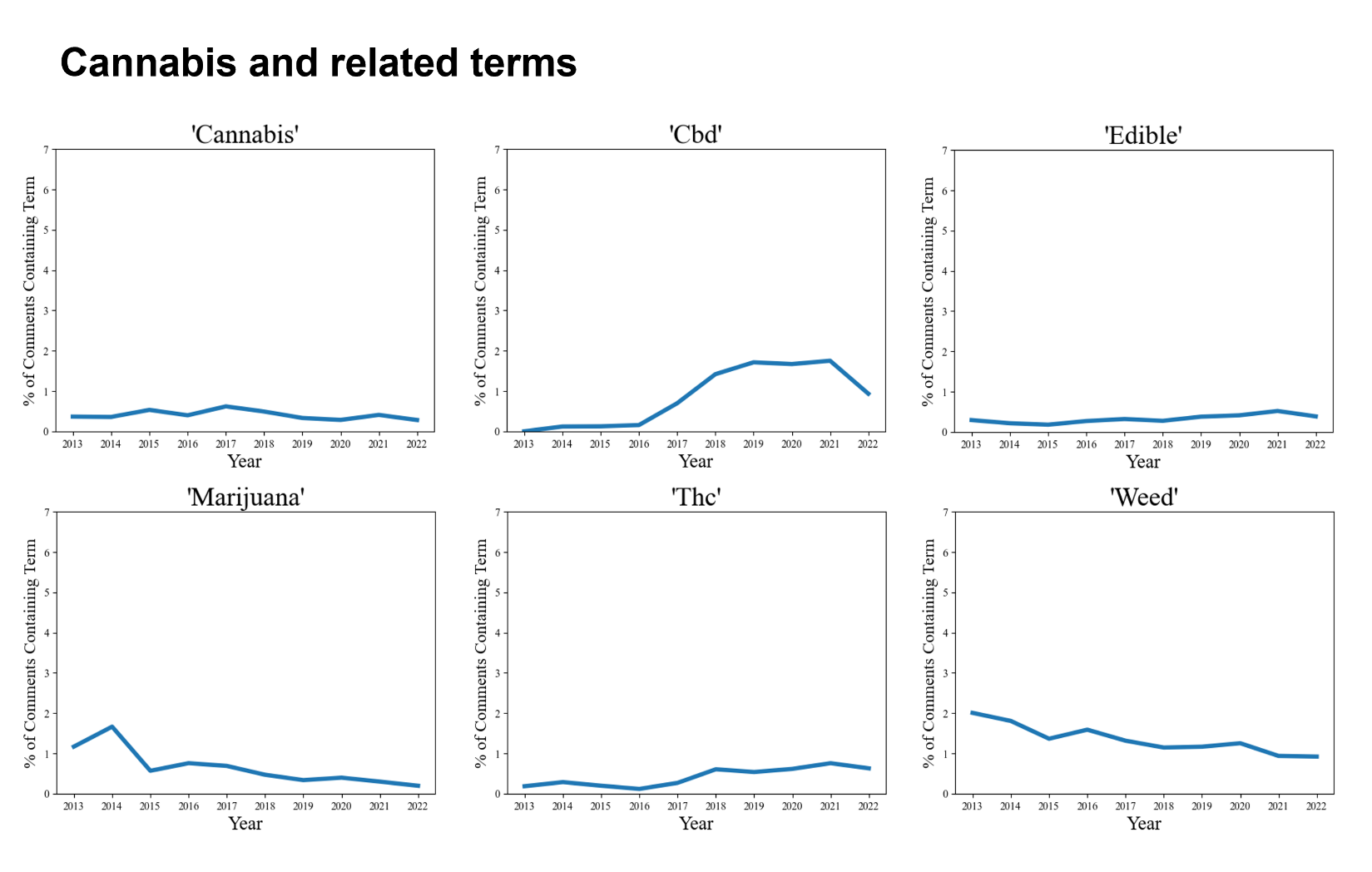
**

**
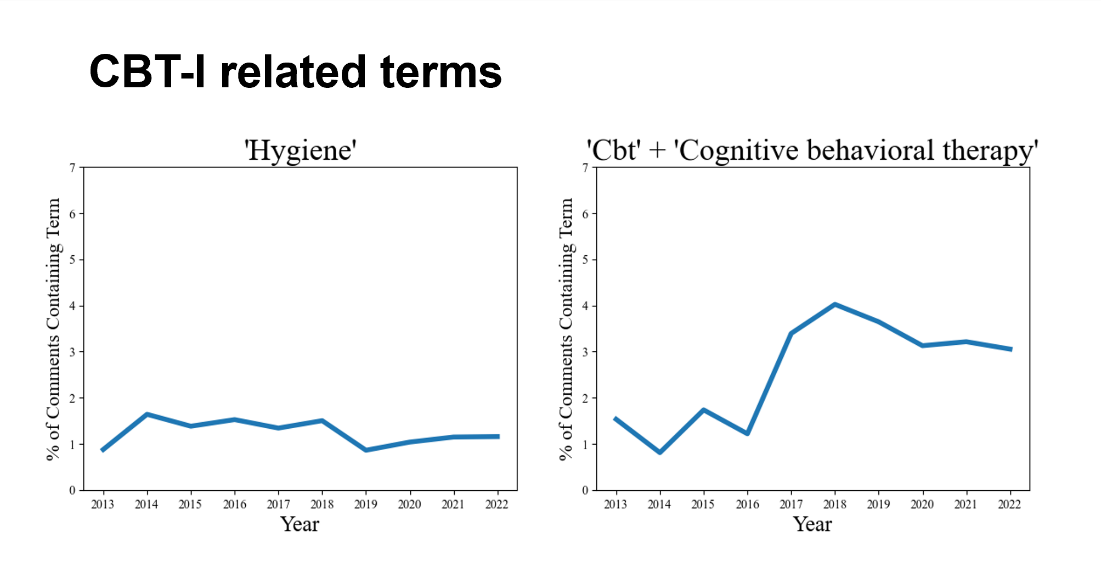
**

**
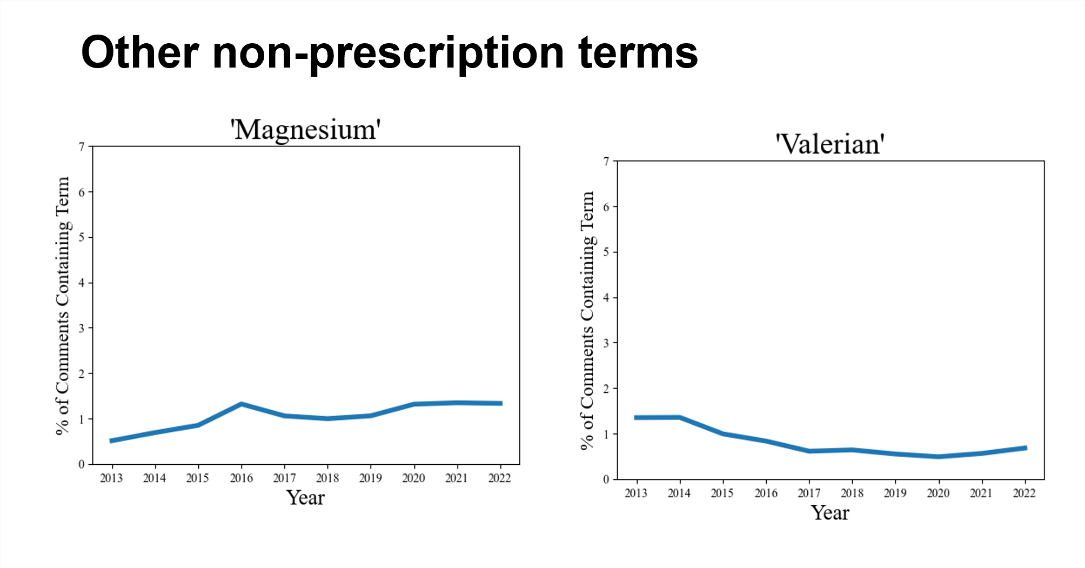
**

**Figure S3***.* Trends in treatment terms in r/insomnia Reddit discussion board over time, grouped by type of treatment.
